# Effects of febuxostat on markers of endothelial dysfunction and renal progression in patients with chronic kidney disease

**DOI:** 10.1038/s41598-023-40767-5

**Published:** 2023-08-18

**Authors:** Naowanit Nata, Nanthawut Ninwisut, Pitchamon Inkong, Ouppatham Supasyndh, Bancha Satirapoj

**Affiliations:** Division of Nephrology, Department of Medicine, Phramongkutklao Hospital and College of Medicine, 315 Rajavithi Road, Bangkok, 10400 Thailand

**Keywords:** Medical research, Nephrology

## Abstract

Hyperuricemia relates to chronic kidney disease (CKD) progression and impaired endothelial function. Febuxostat is potent and effective for decreasing serum uric acid levels. Information for the effect of febuxostat treatment on markers of endothelial dysfunction and renal injury among patients with CKD remains limited. A total of 84 patients with CKD stages III-IV with asymptomatic hyperuricemia were randomly assigned to either the febuxostat (40 mg/day, N = 42) or the matching control (N = 42) group for 8 weeks. Serum asymmetric dimethylarginine (ADMA), estimated glomerular filtration rate (eGFR), urine albumin, high sensitivity C-reactive protein (hs-CRP), ankle brachial index (ABI) and serum uric acid were measured at baseline and at the end of study. Febuxostat administration significantly reduced the serum uric acid concentration among patients with CKD when compared with control [− 3.40 (95% CI − 4.19 to − 2.62) vs. − 0.35 (95% CI − 0.76 to 0.06) mg/dL; P < 0.001, respectively). No significant difference in the changes in serum ADMA, hs-CRP, eGFR and albuminuria was identified between the two groups. Subgroup analysis among patients with decreased serum uric acid after febuxostat, the estimated GFR change between the febuxostat and the control group showed significant difference at 8 weeks (2.01 (95% CI 0.31 to 3.7) vs. 0.04 (95% CI − 1.52 to 1.61) mL/min/1.73 m^2^; P = 0.030, respectively). Adverse events specific to febuxostat were not observed. Febuxostat effectively reduced serum uric acid in the CKD population without improving endothelial dysfunction. It was able to preserve renal function in the subgroup of patients with CKD and lower serum uric acid level after treatment.

**Trial registration: **Thai Clinical Trials, TCTR20210224005: 24/022021 http://www.thaiclinicaltrials.org/show/TCTR20210224005.

## Introduction

Chronic kidney disease (CKD) has become an important public health problem and a major risk factor for cardiovascular disease^1^. Risk factors that are identified and treated at an early stage may prevent or slow disease progression. Hyperuricemia accelerates renal progression and microvascular injury leading to endothelial dysfunction and renal arteriolopathy^2,3^. Epidemiologic studies confirmed that increased serum uric levels as an independent risk factor for CKD and cardiovascular disease^4–6^. Treatment of hyperuricemia might pose one strategy to prevent CKD progression and endothelial injury.

A systematic review and meta-analysis demonstrated that xanthine oxidase inhibitors delay the decline of renal function and reduce the risk of cardiovascular events among patients with CKD^7^. Febuxostat is a potent and selective xanthine oxidase inhibitor among subjects with hyperuricemia and gout, including those with mild to moderately impaired renal function^8,9^. However, all-cause mortality and cardiovascular mortality were higher with febuxostat than with allopurinol among patients with gout and major cardiovascular diseases^10^. Low dose febuxostat showed a superior urate-lowering efficacy to that of allopurinol among Asian patients with advanced CKD^11^. An experimental model suggested that febuxostat improved endothelial function, renal inflammation, tubulointerstitial fibrosis and renal vascular damage^12,13^, which might have renal and cardiovascular benefits in the CKD setting. High uric acid also related to endothelial dysfunction independent of traditional cardiovascular risk factors^14^. Strong clinical evidence to support using xanthine oxidase inhibitors to slow CKD progression and vascular injury remains limited^15,16^. Additionally, few studies of febuxostat treatment on renal function and endothelial function have been conducted among patients with advanced CKD and undergoing dialysis^17,18^. To fill this gap, we performed a randomized controlled trial among CKD stages 3 to 4 patients to demonstrate the renal function and endothelial function after febuxostat treatment.

## Methods

**Figure 1:**
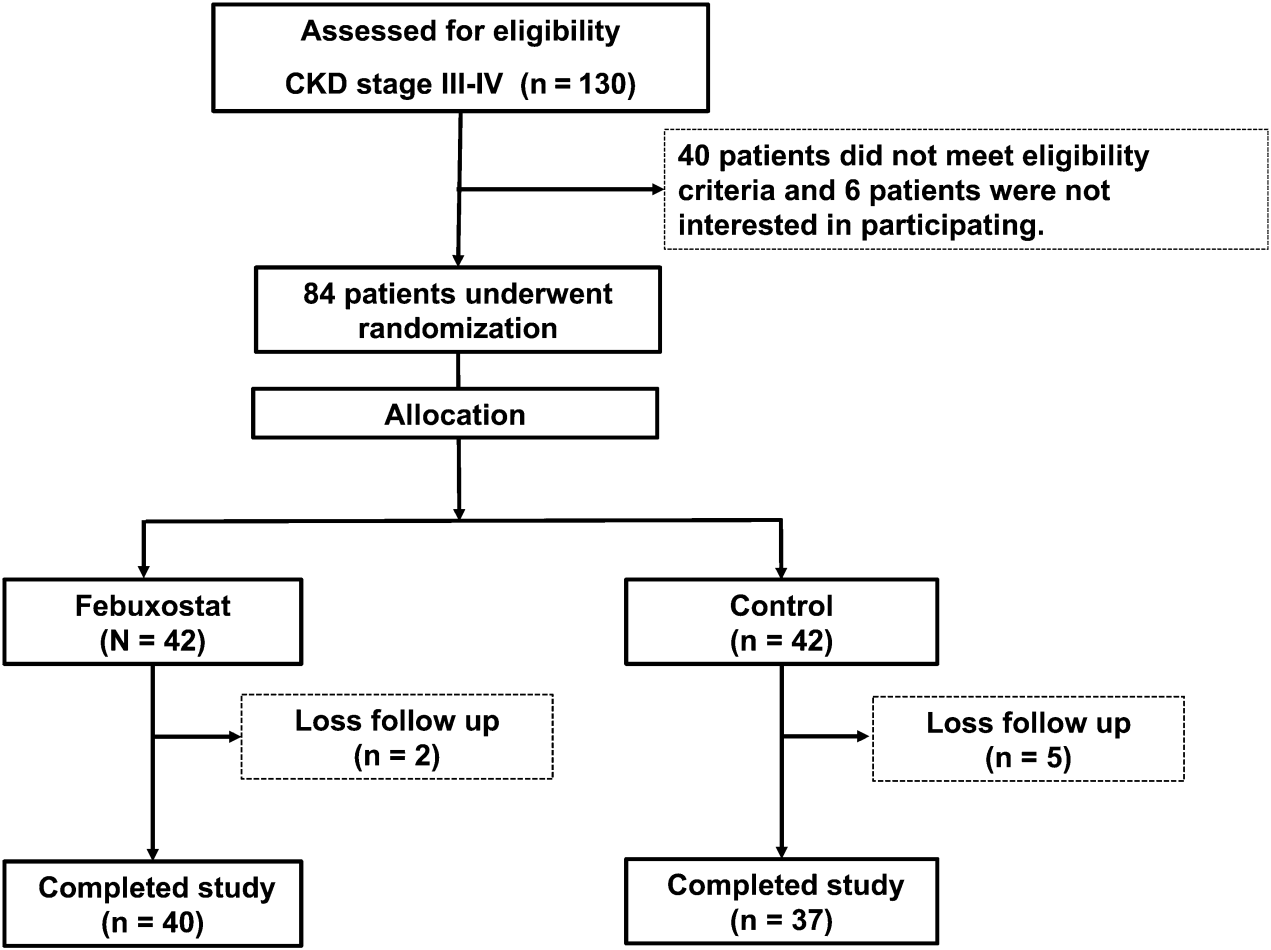
Flow chart of study.

The study comprised a randomized controlled trial comparing the efficacy between febuxostat and control groups. The study was conducted among patients with CKD treated at Phramongkutklao Hospital between 31 January 2018 and 1 February 2019, with all subjects selected using inclusion criteria. Patients were randomized in blocks of four and allocation concealment, then divided in two groups as shown in Fig. 1. Group 1 comprised the group receiving febuxostat 40 mg daily for 8 weeks. Group 2 comprised the group receiving the standard treatment for CKD. The study complies with the Declaration of Helsinki (1964). The study was registered at the Thai Clinical Trials Registry (TCTR) (TCTR20210224005). The study protocol was approved by the ethics committee of the Institute Review Board at the Royal Thai Army Medical Department (IRB Number R048h/61). Written informed consent was obtained from all subjects.

The inclusion criteria included patients with CKD stages III to IV with asymptomatic hyperuricemia, age of 18 years or older, stable dose of all medications including ACEIs or ARBs for blood pressure control at least three months before enrolling and stable renal function within three months before the study. The exclusion criteria comprised active infections, acute kidney injury, advanced malignancy, serious disease, history of hypersensitivity to febuxostat and hospitalization within three months. Based on previous study results, to give 80% power with a two-sided significance level of 0.05 to detect a difference in serum asymmetric dimethylarginine (ADMA) as biomarkers of endothelial dysfunction, a sample size of 38 per treatment group was required to meet the superiority criteria between the febuxostat and placebo groups. Assuming a withdrawal rate of 10%, the target number of patients should be at least 42 in each group^17^.

The data collected before and after in this study, contained relevant information on CKD including diagnostic criteria, complications of disease and other underlying diseases. The history of medications including antihypertensive drugs and physical examination were recorded. The laboratory tests including serum uric acid, blood urea nitrogen, creatinine, calculation of estimated glomerular infiltration rate (GFR) using the 2009 Chronic Kidney Disease Epidemiology Collaboration Equation (CKD-EPI), total cholesterol, low-density lipoprotein and high-density lipoprotein were noted. Participants would receive serum testing for ADMA and high sensitivity C-reactive protein (CRP) by enzyme linked immunosorbent assay. Urine albumin creatinine ratio (UACR) was measured by immunonephelometric assay method after receiving intervention. Ankle brachial index (ABI) was evaluated at baseline using an automatic device (VaSera VS-1000, Fukuda Denshi Co. LTD, Tokyo, Japan). ABI reflected the resistance or compliance of the aorta, femoral artery and tibial artery.

The primary outcome was the change of serum ADMA and renal function after eight weeks in the febuxostat group, compared with that of the control group. The secondary outcome was the improving level of UACR after eight weeks. Any adverse events considered to be related to the use of febuxostat were recorded during the study, and any decision was made by the attending physicians during the study. For serious events, febuxostat was discontinued.

### Statistical analysis

Measured values of the results were expressed as mean with standard deviation and median with interquartile range (IQR). Comparisons between means of continuous variables were determined using the Student t test, and Mann–Whitney U test as appropriate and between groups of dichotomous variables using the Chi-square test or Fisher's exact test. Paired-sample t tests and Wilcoxon signed-rank test were used for continuous variables and presented by the relative risk of 95% confidence intervals and median change with IQR. Statistical analyses were performed using SPSS Program for Windows, Version 15 (SPSS Inc, Chicago, IL, USA). A p < 0.05 was considered statistically significant.

### Ethics statement

The Ethics Committee approved the study (approval number IRB Number R048h/61), and the research was performed according to the Declaration of Helsinki. Study participants gave consent before participating.

## Results

A total of 130 patients with CKD stages III to IV were initially screened and 46 were excluded (including 40 patients not meeting the inclusion criteria, and 6 patients who didn’t sign informed consent forms) (Fig. 1). Forty-two patients were selected to take febuxostat 40 mg daily for 8 weeks. The other 42 patients received standard treatment as the control group. Etiologies of CKD in the study patients involved type 2 diabetes (47.6%), hypertension (21.4%), kidney stone (8.3%), chronic glomerulonephritis (9.5%) and unknown etiology (13.1%). Baseline characteristics and laboratory tests between the two groups are shown in Tables 1 and 2. No significant differences were noted in the febuxostat and control groups in terms of baseline characteristics and laboratory tests.

**Table 1:** Baseline characteristics.

Variables	Febuxostat (N = 42)	Control (N = 42)	P value
Male, n (%)	28 (70)	28 (63.6)	0.537
Age (years)	68.0 ± 15.6	65.4 ± 15.1	0.437
Systolic blood pressure (mmHg)	134.4 ± 12.6	133.8 ± 15.5	0.851
Diastolic blood pressure (mmHg)	75.3 ± 13.5	73.5 ± 10.0	0.543
Right ankle brachial index	1.1 ± 0.2	1.0 ± 0.2	0.093
Left ankle brachial index	1.0 ± 0.2	1.0 ± 0.1	0.170
Etiology of kidney disease (N, %)			
Type 2 diabetes	22 (52.3)	18 (42.8)	0.210
Hypertension	9 (21.4)	9 (21.4)	0.731
Kidney stone	3 (7.1)	4 (9.5)	0.467
Chronic glomerulonephritis	3 (7.1)	5 (11.9)	0.106
Others	5 (11.9)	6 (14.2)	0.872
Anti-hypertensive drugs (N, %)			
Angiotensin II receptor blocker	18(15)	15(31.8)	0.170
Angiotensin converting enzyme inhibitor	3(7.5)	6(13.6)	0.364
Beta blocker	11(27.5)	18(41.9)	0.170
Calcium channel blocker	23(57.5)	26(59.1)	0.883
Furosemides	10(25)	9(20.5)	0.619
Methyldopa	1(2.5)	2(4.5)	0.614
Alpha blockers	10(25)	13(29.5)	0.641

Data in the table are shown with mean ± standard deviation and percentages.

**Table 2:** Baseline laboratory data.

Variables	Febuxostat (N = 42)	Control (N = 42)	P value
Serum uric acid (mg/dL)	8.9 ± 1.35	8.39 ± 1.37	0.094
Serum asymmetric dimethylarginine (umol/L)	0.90 ± 0.47	0.89 ± 0.44	0.788
High sensitivity C-reactive protein (mg/L)	2.6 (1.08, 4.83)	1.61 (0.9, 3.31)	0.258
Urine albumin creatinine ratio (mg/gCr)	331.4 (14.1, 1534)	229.3 (48, 762)	0.716
Serum creatinine (mg/dL)	2.17 ± 0.75	2.31 ± 0.86	0.440
Estimated GFR (mL/min/1.73 m^2^)	31.22 ± 10.23	31.82 ± 14.35	0.825
LDL-cholesterol (mg/dL)	91.63 ± 32.69	94.92 ± 35.18	0.661
Serum bicarbonate (mEq/L)	24.6 ± 2.99	23.89 ± 2.17	0.212
Serum calcium (mg/dL)	8.95 ± 1.51	9.22 ± 0.6	0.285
Serum phosphate (mg/dL)	3.26 ± 0.56	3.34 ± 0.57	0.492
Serum albumin (g/dL)	4.02 ± 0.52	4.22 ± 0.37	0.045
AST (U/L)	20.24 ± 6.43	21.53 ± 7.52	0.403
ALT (U/L)	20.07 ± 11.16	18.53 ± 8.81	0.489

Data in the table are shown with mean ± standard deviation and median with interquartile range (IQR).

*GFR* glomerular filtration rate, *LDL* low density lipoprotein cholesterol.

**Figure 2:**
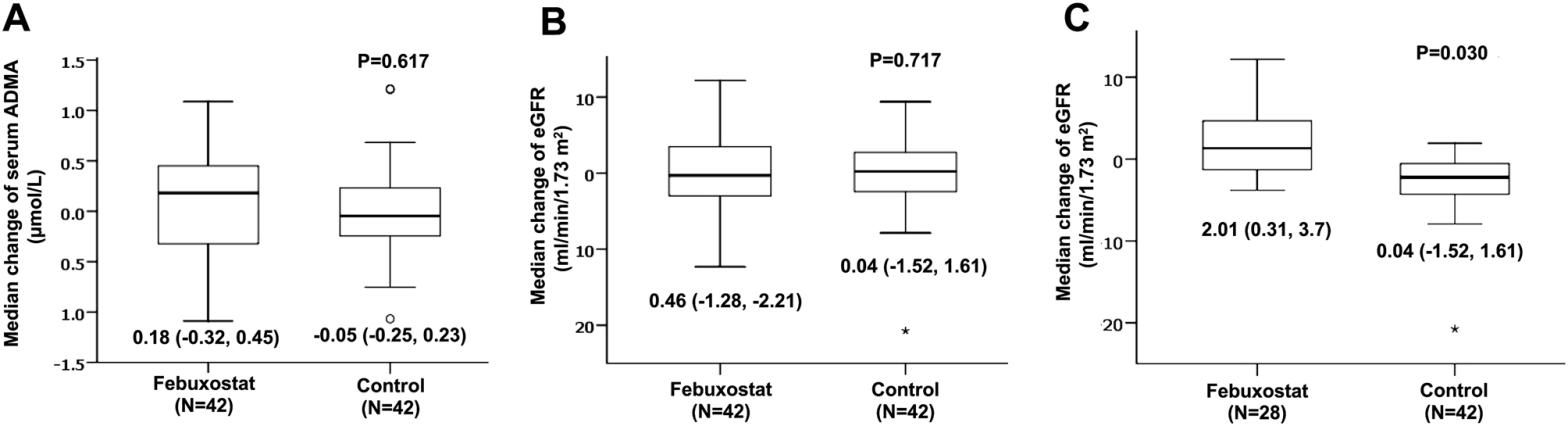
Change of endothelial function and renal function after treatment. Box-and-whisker-plot diagram shows the (**A**) median change of serum asymmetric dimethylarginine, (**B**) median change of estimated GFR in all patients and (**C**) median change of estimated GFR in subgroup of febuxostat patients with lower serum uric acid after treatment.

At eight weeks, significant differences were found on mean change of serum uric acid level between the febuxostat and control groups (− 3.4 (95% CI − 4.19 to − 2.62) vs. − 0.35 (95% CI − 0.76 to 0.06) mg/dL, P < 0.001, respectively). However, no significant differences were found on change of renal function, urine albumin, serum ADMA, high sensitivity-CRP and ABI between the two groups as shown in Table 3 and Fig. 2. After additional analysis in a group of 70 patients with decreased serum uric acid after febuxostat treatment compared with the control group; mean estimated GFR in the febuxostat group showed a significant increase from 30.6 ± 10.1 to 32.7 ± 12.5 mL/min/1.73 m^2^ (P = 0.022). In the control group, mean estimated GFR showed no significant change from a baseline of 31.3 ± 15.4 to 31.4 ± 13.1 mL/min/1.73 m^2^ (P = 0.802). The estimated GFR change between the febuxostat and the control groups significantly differed at eight weeks (2.01 (95% CI 0.31 to 3.7) vs. 0.04 (95% CI − 1.52 to 1.61) mL/min/1.73 m^2^; P = 0.030, respectively), as shown in Fig. 2C.

**Table 3:** Change of variables after 8 weeks of treatment.

Variables	Febuxostat (N = 42)	Control (N = 42)	P value
Serum uric acid (mg/dL)			
Baseline	8.90 ± 1.35	8.39 ± 1.37	0.094
At 8-week	5.49 ± 2.23	8.03 ± 1.89	< 0.001
Mean change with 95%CI	− 3.4 (− 4.19, − 2.62)	− 0.35 (− 0.76, 0.06)	< 0.001
Estimated GFR (mL/min/1.73 m2)			
Baseline	31.22 ± 10.23	31.82 ± 14.35	0.825
At 8-week	31.47 ± 12.64	32.26 ± 15.59	0.802
Mean change with 95%CI	0.46 (− 1.28, 2.21)	0.44 (− 1.52, 1.61)	0.717
Urine albumin creatinine ratio (mg/gCr)			
Baseline	331.4 (14.1, 1534)	229.3 (48, 762)	0.716
At 8-week	396 (73.3, 1849)	363 (59, 1438)	0.838
Median change (IQR)	0 (− 109, 140.1)	0.1 (− 117, 45.6)	0.725
Serum asymmetric dimethylarginine (umol/L)			
Baseline	0.90 ± 0.47	0.89 ± 0.44	0.788
At 8-week	1.04 ± 0.42	0.88 ± 0.48	0.131
Mean change with 95%CI	0.18 (− 0.32, 0.45)	− 0.05 (− 0.25, 0.23)	0.617
Serum high sensitivity C-reactive protein (mg/L)			
Baseline	2.6 (1.08, 4.83)	1.61 (0.9, 3.31)	0.258
At 8-week	2.21 (1.32, 4.19)	2.04 (0.86, 4.07)	0.899
Median change (IQR)	− 0.15 (− 1.59, 0.8)	− 0.18 (− 0.64, 0.82)	0.842
LDL-cholesterol (mg/dL)			
Baseline	94.92 ± 35.18	91.63 ± 32.69	0.661
At 8-week	92.5 ± 31.82	90.93 ± 31.22	0.826
Mean change with 95%CI	− 3.4 (− 11.87, 5.08)	0.18 (− 7.94, 8.3)	0.539
Ankle brachial index: Right			
Baseline	1.09 (1, 1.19)	1.06 (0.98, 1.12)	0.093
At 8-week	1.09 (1.03, 1.17)	1.07 (1, 1.14)	0.245
Median change (IQR)	0.01 (− 0.07, 0.06)	0 (− 0.04, 0.05)	0.734
Ankle brachial index: Left			
Baseline	1.07 (0.99, 1.14)	1.03 (0.97, 1.1)	0.170
At 8-week	1.06 (1.01, 1,12)	1.03 (0.96, 1.12)	0.515
Median change (IQR)	− 0.01 (− 0.07, 0.04)	0 (− 3.61, 7.69)	0.287

Data in the table are shown with mean ± standard deviation, mean with 95%CI, and median with interquartile range (IQR).

*GFR* glomerular filtration rate, *LDL* low density lipoprotein cholesterol.

Very few adverse events were directly related to febuxostat during the study period (4.7% (2 of 42 patients). One male patient had spontaneous remission of skin rash and another patient had mild abnormal liver function. Our studied patients did not experience any serious side effects of febuxostat during the study, such as major allergic reactions, severe hepatitis or major cardiovascular event.

## Discussion

In the study, febuxostat at 40 mg daily showed significantly reduced serum uric acid level among patients with CKD stages III to IV and without serious adverse events documented, consistent with a related study^11^. However, no significant improvements were observed regarding renal function, albuminuria and endothelial dysfunction after short term febuxostat treatment.

Febuxostat constitutes a nonpurine selective xanthine oxidoreductase inhibitor and a high potency for uric acid lowering agent, but limited studies have addressed the efficacy of febuxostat among patients with advanced CKD^8,9^. Recent studies of febuxostat among patients with advanced CKD showed that more than 70 to 80% of these patients could achieve the serum uric acid target less than 6 mg/dL^11,19^.

Observational studies suggest that uric acid is a potential risk factor for developing CKD and cardiovascular progression^4–6,20^. Proposed mechanisms of uric acid and CKD progression induce oxidative stress, renin-angiotensin system activation, renal epithelial-mesenchymal transition and endothelial dysfunction resulting in developing renal arteriolosclerosis and cardiometabolic disease^21^. Experimental models also indicated that lower serum uric acid with allopurinol or febuxostat improved kidney injury^22^. Data exist examining the effects of uric acid-lowering therapy on improved renal outcomes from observational studies, small clinical controlled trials and meta-analysis^7,23^. Currently, the effect of uric acid-lowering therapy on renal function remains controversial. Recently, two large randomized control trials from the FEATHER study indicated that febuxostat did not slow estimated GFR decline among patients with early CKD and asymptomatic hyperuricemia^15^. The FREED study also indicated that febuxostat did not improve change of estimated GFR among elderly patients with hyperuricemia^24^, consistent with our findings. However, the FREED study indicated that febuxostat exhibited a large reduction of serum uric acid levels, and its effect was associated with reduced renal impairment^24^. Therefore, it exerts a reasonably strong serum uric acid-lowering effect of febuxostat on organ protection. However, the main effects of febuxostat on renal outcome might depend on uric acid lowering effects. Our finding also showed that estimated GFR improved only patients with significantly lower serum uric acid after intervention. Therefore, major effects of febuxostat on renal outcome might be uric acid dependent effects.

Endothelial dysfunction is an initial phase in the vascular damage and atherosclerotic process. Hyperuricemia and advanced CKD especially in a dialysis population are related to endothelial dysfunction by impairing the nitric oxide bioavailability and markers of endothelial dysfunction are associated with stage of CKD^25^. Xanthine oxidase inhibitors produce benefits concerning endothelial function by reducing oxidative stress^26^. Several randomized controlled studies supported that low dose febuxostat for four to eight weeks improved serum uric acid, oxidative stress, endothelial dysfunction and inflammation markers among patients undergoing hemodialysis^17,18,27^. On the other hand, our study did not confirm a beneficial effect of lowering the markers of endothelial dysfunction (serum ADMA), systemic inflammation (high sensitivity-CRP) and vascular stiffness (ABI) after eight weeks of febuxostat treatments among patients with CKD stages III to IV patients. Low levels of inflammation and endothelial dysfunction at baseline in our CKD population might have been the major effect on treatment outcomes.

Limitations encountered in this study included a relatively small sample size; one only half of patients were treated with ACEIs or ARBs and only 3.6% of patients were given sodium–glucose cotransporter 2 inhibitors. The observational period of the present study lasted eight weeks which was relatively short to determine renal outcome; thus, further study with a longer follow-up was needed. We had measurement of biomarkers for endothelial dysfunction (serum ADMA, high sensitivity-CRP and ABI measurement) and were unable to longitudinally assess the relationship between changes in endothelial dysfunction and renal function. Therefore, the long term side effects of treatment are needed to be further investigated.

## Conclusion

In summary, febuxostat effectively reduced serum uric acid among patients with CKD without improvement of endothelial dysfunction and renal function. It was able to preserve renal function in a subgroup of patients with CKD and lower serum uric acid level after treatment. Further study is needed to determine the long term effects of febuxostat on endothelial function and renal progression among patients with advanced CKD.

## Data Availability

The datasets generated and analyzed during the current study can be made available upon request through the corresponding author (BS).
